# Phage therapy: a revolutionary shift in the management of bacterial infections, pioneering new horizons in clinical practice, and reimagining the arsenal against microbial pathogens

**DOI:** 10.3389/fmed.2023.1209782

**Published:** 2023-10-19

**Authors:** Subhash Lal Karn, Mayank Gangwar, Rajesh Kumar, Satyanam Kumar Bhartiya, Gopal Nath

**Affiliations:** ^1^Department of Microbiology, Faculty of Medicine, Institute of Medical Sciences, Banaras Hindu University, Varanasi, India; ^2^Department of General Surgery, Faculty of Medicine, Institute of Medical Sciences, Banaras Hindu University, Varanasi, India

**Keywords:** bacteriophage, veterinary medicine, preclinical AND clinical trials, immune response, treatment challenges

## Abstract

The recent approval of experimental phage therapies by the FDA and other regulatory bodies with expanded access in cases in the United States and other nations caught the attention of the media and the general public, generating enthusiasm for phage therapy. It started to alter the situation so that more medical professionals are willing to use phage therapies with conventional antibiotics. However, more study is required to fully comprehend phage therapy’s potential advantages and restrictions, which is still a relatively new field in medicine. It shows promise, nevertheless, as a secure and prosperous substitute for antibiotics when treating bacterial illnesses in animals and humans. Because of their uniqueness, phage disinfection is excellent for ready-to-eat (RTE) foods like milk, vegetables, and meat products. The traditional farm-to-fork method can be used throughout the food chain to employ bacteriophages to prevent food infections at all production stages. Phage therapy improves clinical outcomes in animal models and lowers bacterial burdens in numerous preclinical investigations. The potential of phage resistance and the need to make sure that enough phages are delivered to the infection site are obstacles to employing phages *in vivo*. However, according to preclinical studies, phages appear to be a promising alternative to antibiotics for treating bacterial infections *in vivo*. Phage therapy used with compassion (a profound understanding of and empathy for another’s suffering) has recently grown with many case reports of supposedly treated patients and clinical trials. This review summarizes the knowledge on the uses of phages in various fields, such as the food industry, preclinical research, and clinical settings. It also includes a list of FDA-approved bacteriophage-based products, commercial phage products, and a global list of companies that use phages for therapeutic purposes.

## Introduction

If the crisis of antimicrobial resistance is not addressed, it is anticipated that by 2050, the societal and financial costs will total US$100 trillion, resulting in 70,000 annual deaths (1). This number, although controversial, still emphasizes the severe problem we face regarding therapeutic alternatives for multidrug-resistant (MDR) bacterial infections (2, 3). Significant health risks are posed by pathogens, including methicillin-resistant *Staphylococcus aureus* (MRSA) and multi-resistant *Pseudomonas aeruginosa* (MDR-PA). The World Health Organization (WHO) recently published a list of MDR-priority pathogens and called for more research into antimicrobial resistance (4). Equally concerning is the requirement for rapid development of new antibiotics to replace older ones that are losing their efficacy. As a result, many researchers and clinicians are looking at bacteriophage as the most potential substitute for or adjunct to antibiotics to treat bacterial infections in the face of rising antibacterial resistance. Bacteriophages are viruses that uniquely and specifically target and eliminate bacteria. They work cooperatively in microbiological ecosystems in the human body and the environment and do not harm mammalian cells. As natural biological regulators, bacteriophages integrate into the One Health Strategy for animals, humans, and the environment (5). The first phages to be identified were phages against *Escherichia coli*, *Shigella dysenteriae*, and *Vibrio cholerae* (6, 7). Although there are several reports on phage treatment, they are uncontrolled or anecdotal and must adhere to the standards of contemporary evidence-based medicine (8). After the discovery of antibiotics, phages ceased being used as antibacterial agents in Western countries. Given our present in-depth understanding of the biology of bacteriophages, which is crucial in supporting developments in molecular biology, the odds of success the second time around are significantly greater. However, evidence from clinical and animal model studies strongly suggests that phage treatment is secure and may be advantageous (9–11).

This review offers recommendations for clinicians considering experimental phage treatment based on a thorough literature evaluation due to knowledge gaps. The study also intends to give an evidence-based assessment of the situations in which this experimental therapy may be considered and to acquaint clinicians with phage therapy’s preclinical and clinical usage.

## Bacteriophages vs antibiotics

### Enzybiotics

According to Veiga-Crespo et al. (12), the term “enzybiotics” refers to phages, viruses that target and lyse bacteria and may potentially aid in the treatment of bacterial infections. These can be phage-encoded lytic enzymes, such as lysins, and extracellular polymeric substance (EPS) depolymerase. Even though many EPS depolymerases and certain lysins are connected to virion particles (13), most lysins are endolysins, which are “from-within cell-wall degrading enzymes.” Enzybiotics, however, are added from outside after purification. Gram-positive bacteria’s peptidoglycan is not shielded by an outer membrane, making it susceptible to phage lysins. In order to cross the outer membrane barrier in Gram-negative bacteria, phage lysins often must be altered. Fusion proteins that combine natural lysin with an antibacterial peptide might accomplish this. According to the study by Yang et al. (14), one of these constructs, PlyA, showed good efficacy against *A. baumannii* and *P. aeruginosa* and growing cultures but not against cells in the stationary phase unless combined with agents that permeabilize the outer membrane. Due to a lack of awareness and comprehension, engineered bacteriophages could pose a problem in terms of public acceptance and regulatory approvals of engineered bacteriophages could delay their usage (15). In contrast to antibiotics, phages have genomes and can proliferate while parasitizing their host; hence, engineering them could cause additional problems and necessitate the addition of legal clauses addressing the social issues associated with genetic modifications. Additionally, when lysins are used via intravenous administration, neutralizing antibodies are produced, reducing their antibacterial efficacy after multiple administrations (16). However, few endolysin immunogenicity studies have shown that immune serum does slow but is not sufficient to block the antibacterial ability of endolysin (17–19). Since they have a short plasma half-life, are immunogenic and potentially toxic, cause an inflammatory reaction to bacterial debris, and are ineffective in lysing intracellular bacteria, their use as an antibacterial agent in human treatment raises concerns (20).

## Delivery system for phage and Endolysin

Despite therapeutic potential of phages and endolysins, these alternative agents must overcome various practical challenges posed by the host system, such as limited bioavailability, loss of action, non-targeted delivery, rapid clearance by the reticuloendothelial system, and antibody-mediated inactivation (21).

Numerous methods for encapsulating phage and endolysin have been discovered recently (21). These delivery methods treat acute and chronic infections in animal models by altering host immunological response to therapeutic entities and increasing pharmacokinetic parameters (22). Many phage encapsulation studies have investigated the possibilities of different drug delivery systems, primarily natural polymers, synthetic polymers, liposomes, and electrospun fibers. GIT infections have been thoroughly researched as a potential polymeric phage encapsulation therapy target. These polymers shield phages from harsh acidic environments that may otherwise result in phage inactivation or loss of phage titer. In addition to safeguarding phages from harsh environments, these polymeric encapsulation materials also give permeability to the mucosal membrane, where bacterium pathogens may live, and get protected from digestive enzymes and bile fluids (23). According to Gondil et al., phage delivery systems are very effective when an infection is still in its early stages and can be effectively eliminated with just one dose of a phage formulation (24). However, late administration of phage formulation necessitates concomitant antibiotic administration or multiple doses of phage formulation to prevent the spread of infection. Different delivery systems of bacteriophage and endolysin are mentioned in Table 1.

**Table 1:** Showing phage delivery systems.

Delivery system	Types	Protective Properties
Stabilized dry phage preparation (powders)	Lyophilized Phages Spray drying	The biotherapeutic material is still active after lyophilization, enabling long-term storage. The particle sizes produced range from nanometers to micrometers. Particle sizes between 1 and 5 mm are typically produced via spray drying. Such nano- or microparticle manufacturing enables the creation of phage powders that are simple to administer for treating respiratory infections because distribution via inhalers enables effective nebulization (25–28).
Encapsulation	Liposomes Transferosome Hydrogels Electrospinning	Liposomes shield the cargo from enzymatic attack, hydrolysis (low pH), and inactivation by immune system components. They can penetrate bacterial biofilms. It also helps the retention of phages at the site of infection. Oral liposome formulations are the most effective method for treating gastrointestinal illnesses (21, 29, 30). With better skin penetration and higher soft tissue protection than a free-phage cocktail, transferosomes—liposomes incorporating detergent are effective delivery systems (31). Phage hydrogel encapsulation demonstrated high antibacterial activity in an alginate encapsulation and was successfully shielded from the acidic stomach pH. A phage cocktail in CaCO_3_ demonstrated higher antibacterial activity (32). It is possible to produce a wide variety of materials. A fiber-encapsulated phage can be quickly deposited into other substances (33).
Immobilization with fibers	Bandages and dressings	The topical delivery of phages in the form of bandages and dressings for wounds and packing materials with acidic pH and antibacterial enzymes is made possible by surface immobilization of the phages (34).

## Bacteriophage and its applications

**Figure 1:**
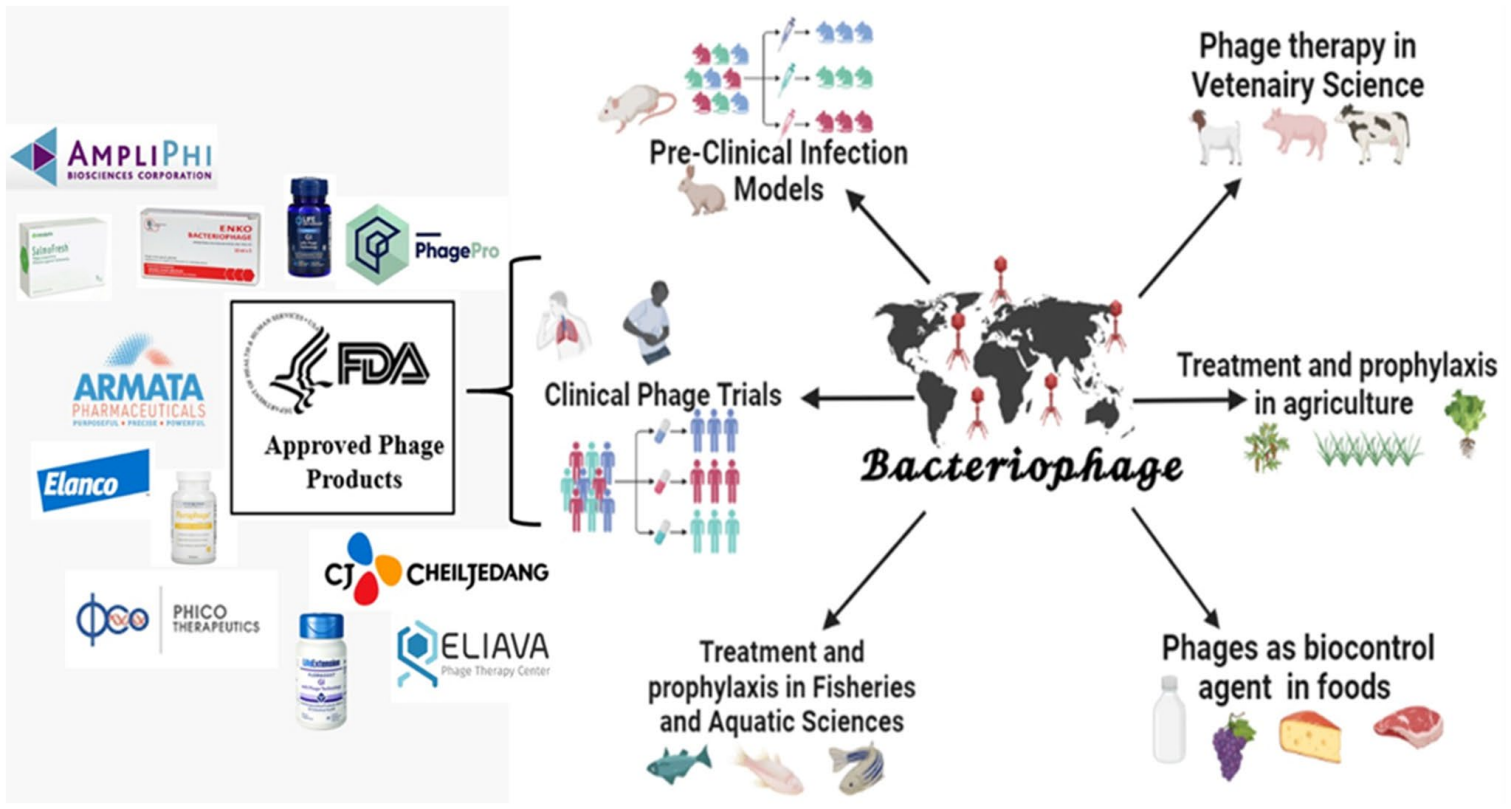
Applications of bacteriophages and its FDA-approved products (created by the help of bio-render tool).

Bacteriophages have been used and reported worldwide in various valuable applications. Figure 1: graphically represents the use of phage in clinical, preclinical, agriculture, fishery, and food industries. In addition, the therapeutic efficacy of the phage has been proven using preclinical and clinical settings. Based on these results, many pharmaceutical companies are still running clinical trials using bacteriophage products. However, the FDA has approved various phage products competing with the existing antimicrobials.

## Bacteriophages in preclinical studies

Recent years have seen a resurgence in interest in phage treatment, which uses bacteriophages to treat bacterial illnesses. This is because antibiotic-resistant bacteria are becoming more common. However, before going on to clinical trials in people, preclinical phage application involves evaluating the tolerance and efficacy of phage therapy in animal models.

Preclinical trials of phage therapy in animals typically involve infecting the animals with a specific bacterial pathogen and then administering phages to see if they can reduce bacterial loads and improve clinical outcomes. In addition, the animals are monitored for signs of toxicity or adverse reactions to the phages. Phage therapy improves clinical outcomes in animal models and lowers bacterial burdens in numerous preclinical studies. Nevertheless, there are challenges to using phages *in vivo*, such as the risk of phage resistance and the requirement to ensure that enough phages are delivered to the infection site.

### Pharmacokinetics and pharmacodynamics of bacteriophages

Lytic phages, unlike conventional antibiotics, are unique biological agents capable of replicating within susceptible bacteria. They present complex pharmacokinetic (PK) profiles that encompass various facets, including absorption, distribution, metabolism, and elimination. The bedrock of phage dosage determination lies in the quantity of administered phage particles. Often, the choice of assays for quantifying phages in dosing solutions and biological samples, like blood and urine, is overlooked (35).

The primary method for quantifying phages involves counting visible plaques on agar plates containing susceptible bacteria. However, this approach may not encompass the entire phage population, as it only considers those capable of causing extensive lysis and resulting in visible plaques. Additionally, the concept of “efficiency of plating,” expressed as the ratio of plaque-forming units (pfu) of phages on the target bacterial strain relative to a reference strain, is integral to phage quantification. Using a bacterial host strain with lower plating efficiency can lead to underestimations, necessitating adjustments for an accurate count of infective phages against the target strain.

In the clinical arena, quantitative PCR traditionally monitors the kinetics of viral load for human viruses like HIV, CMV, Hepatitis B & C, and SARS-Cov-2, aiding in disease assessment and evaluating the efficacy of antiviral therapies. Recently, quantitative PCR-based assays have been applied to monitor phage PK in patients with severe bacterial infections undergoing intravenous adjunctive phage therapy. However, these assays face limitations in distinguishing infective phages from non-infective ones or phage DNA/RNA fragments. Therefore, further research is imperative to establish correlations between PCR-based assays and therapeutic outcomes. In conclusion, the pressing need for sensitive and validated methods of quantifying phages remains a paramount concern in the field of phage PK/PD research.

Phage therapy, unlike antibiotics relying on the widely accepted minimum inhibitory concentration (MIC) for evaluating pharmacodynamics (PD), grapples with standardizing antibacterial activity assessment. Traditional approaches encompass agar-based spot tests and efficiency of plating assays, while fluid environments like broth employ planktonic killing assays to assess phage effectiveness. These different testing methods yield divergent results in terms of the host range for the same phage; direct spot tests exhibit the broadest coverage, followed by efficiency of plating and broth killing assays. This variability arises from distinct killing kinetics and the emergence of phage resistance under different testing conditions. Evaluating the strengths and limitations of each method comprehensively is crucial for enhancing phage efficacy assessment (35, 36).

Recognizing that the dynamics of phage resistance observed in controlled laboratory settings may not necessarily mirror what transpires in animals or humans is essential. Initiatives like Clinical Phage Microbiology aim to provide guidance for clinical decision-making, underscoring the significance of incorporating knowledge related to phage pharmacokinetics and pharmacodynamics (PK/PD) to optimize therapy. Developing a robust phage PK/PD framework requires the standardization of PD parameters and the establishment of dependable measurement methods to inform treatment protocols. This endeavor holds immense promise for advancing the field of phage therapy and its application in clinical practice.

However, there are only a few studies that cover the pharmacology of bacteriophage therapy, and even fewer that focus on the pharmacokinetics of phage therapy. Pharmacology focuses on how drugs interact with the body; it is further divided into pharmacokinetics, which evaluates how the body impacts drugs, and pharmacodynamics, which examines how drugs impact the body. In addition, the dosage quantity of a given phage cocktail was crucial for achieving enhanced pharmacokinetics; the high dosage. As it is crucial to produce the pharmacodynamic effects of the treatment, pharmacokinetics explains how well a drug can accumulate in the locality of the targeted tissues and is summarized in absorption, distribution, metabolism, and excretion (36).

According to Abedon et al. (35), drug dilution causes drug densities to decrease during both absorption and distribution, which may result in an increase in drug density in the targeted bodily organ. Different medication delivery methods are used depending on the pharmacokinetics. A variety of criteria are taken into consideration when choosing a drug’s delivery method, including the target tissue, the drug’s sensitivity to body enzymes, the patient’s convenience, and immunity. The formulation of the phage is yet another crucial factor in establishing efficient pharmacokinetics. The stability of phages is improved by using various formulation techniques. When treating various infections in both animal models and humans, it is important to take into account the different pharmacokinetics concepts that are necessary for the phage to penetrate the target bacteria, the achievement of an adequate phage concentration in the target’s locality, and an ample antibacterial response against the target.

Since 2010, all relevant research results regarding phages *in vivo* in preclinical studies have been compiled (Table 2). The published reports are systematically presented under the headings *viz.* infection syndrome, the animal used in the study, route of phage administration, microbial organism, clinical outcome, and findings, and reported adverse events, if any. Overall, preclinical studies on phages as an alternative to antibiotics for treating bacterial infections show promise *in vivo*. However, more investigation is required to completely comprehend the safety and effectiveness of phage therapy and identify the best dosage and delivery techniques for various bacterial infections and animal species (Table 3).

**Table 2:** Potential advantages of bacteriophage treatment over antibiotic treatment.

Characteristics features	Phage treatment	Antibiotics treatment	References
Specificity	Highly specific	Broad range of action	(37)
Effect on Normal flora	Minimal effect on normal flora with no dysbiosis and chances of developing secondary infections	Possess a broad spectrum of activities likely to affect microbial balance in patients and that are likely to generate severe secondary infections.	(38, 39)
Toxicity	Almost non-toxic	varying degrees of toxicity that range from mild to severe	(39–41)
Biofilm Penetration	Ability to penetrate effectively	cannot penetrate unless applied in large doses	(42–48)
Possibility of resistance	Reduced potential to induce bacterial resistance	High possibility of resistance	(39, 49–51)
Replication at the site of infection	Replicate at the infection site, making them accessible where they are most needed.	They do not always concentrate at the site of infection; instead, they are metabolized and excreted from the body.	(40, 50–52)
Adaptation to bacterial mutation	Can rapidly adapt to bacterial mutation	Unable to adjust to bacterial mutation	(40, 50)
Cross-resistance	Lack of cross-resistance to phages	Resistance mechanisms can also impact the effectiveness of various classes of antibiotics to a specific family of antibiotics.	(38, 39, 41, 50, 53, 54)
Dosage	Sometimes require multiple doses	Repeat doses are necessary	(40, 51)
Environment impact	Low impact on the environment	High environment impact	(40, 49, 55)
Effect on inflammatory responses	Possible effect on the inflammatory response	No effect on the inflammatory response	(56, 57)
Cycle of development	New phages (against phage-resistant bacteria) can rapidly be developed and be accomplished in days or weeks.	The long and expensive development cycle may take several years.	(55, 58)

**Table 3:** A list of recent studies that are pertinent to the use of phages *in vivo* in preclinical trials.

Infection Syndrome	Animal (Species/Strain)	Route of administration	Target bacteria	Clinical outcome	Outcome assessed	Adverse events	Article (references) and Country
Urinary tract infections	Mice (C57BL/6NCrl)	Intraperitoneal	*Cronobacter turicensis*	The kidney’s bacterial load was reduced by 70% after receiving phage (10^11^) PFU/mL without impacting the kidney’s antioxidant status.	Bacterial load	None	Tothova et al. (59) Slovakia
	Mice (Kunming)	Intraperitoneal	*Salmonella enteritidis*	Single phage (10^10^) PFU/mL treatment given an hour after a bacterial challenge prevented 40% of the mice from developing a fatal illness.	Mortality	None	Tang et al. (60) China
Bacteremia	Mice (BALB/c)	Intravenous	*S. enterica serovar* Paratyphi B	Phage administered two weeks after infection was utterly effective in sterilizing the animals; it also showed that phage-resistant bacteria could be excellent vaccines with reduced virulence.	Mortality and bacterial load	None	Capparelli et al. (51) Italy	
	Mice (BALB/c)	Intraperitoneal	*Klebsiella pneumoniae*	The frequency of bacterial mutations was decreased by employing a phage cocktail comprising three lytic phages (GH-K1, GH-K2, and GH-K3) with various but overlapping host strains. Furthermore, phage cocktail treatment saved more animals than single phage treatment.	Mortality	None	Gu et al. (61) China	
	Mice (ICR)		*Pseudomonas aeruginosa*	Using various MOIs, a single phage was injected into immunocompetent and neutropenic mice (1, 10, and 100). All MOIs tested resulted in 80–100% rescue of normal mice. However, the phage did not offer protection to the infected neutropenic mice.	Mortality	None	Tiwari et al. (62) Republic of Korea	
	Mice (ICR)	Intraperitoneal	*Staphylococcus aureus*	Mice exhibiting considerable phage replication throughout time, particularly in the liver and spleen, were administered intraperitoneal phage S13’ six hours after infection. This treatment prevented the development of lung-derived septicaemia and lowered the severity of the infection.	Mortality	None	Takemura Uchiyama et al. (63) Japan	
	Mice (C57BL/6)	Intraperitoneal vs. Oral (Intragastric)	*K. pneumoniae*	A single dosage of NK5 less than 2 × 10^8^ PFU/mL administered intraperitoneally or intragastrically 30 min after *K. pneumoniae* infection prevented animals from dying in a dose-dependent manner. While i.p. injection produced better effects with late phage treatment (30 min), intragastric administration provided more robust protection with early phage administration (6-24 h).	Mortality	None	Hung et al. (64)Taiwan	
	Rat (Sprague Dawley rat pups)	Intraperitoneal or Subcutaneous	*Escherichia coli*	Sepsis and meningitis models were used to assess the therapeutic efficacy of a single dosage of phage (10^8^ PFU/mL) given 7 or 24 h after infection. In these animals, survival was 100 and 50%, respectively.	Mortality	None	Pouillot et al. (65) France	
	Mice (BALB/c)	Intraperitoneal	*Enterococcus faecalis*	A single intraperitoneal phage injection (4 × 10^5^ PFU/) given an hour after the bacterial challenge was enough to prevent all mice from bacteraemia and cause faster bacterial clearance in the blood of protected mice than in unprotected mice. However, phage should be controlled and used appropriately to avoid imbalance in the gut microbiota.	Mortality	None	Cheng et al. (66) China	
	Mice (BALB/c)	Intraperitoneal	*Acinetobacter baumannii*	The study assessed the safety, effectiveness, and delivery strategies of the Abp1 (phage) in treating local and systemic *A. baumannii* infections. When phage was administered to animals right after bacterial infection, they had a 100% survival rate. Abp1 effectiveness is equal to polymyxin B (10 mg/kg).	Mortality	None	Yin et al. (67), China	
	Mice (BALB/c)	Intravenous	*E, coli*	When given within 60 min of bacterial infection, bacteriophage had a 100% curative effect and saved all affected mice’s lives.	Mortality	None	Schneider et al. (68) Hungary	
	Rat (Wistar)	Intramuscular	*P. aeruginosa*	In cases of endocarditis, a single dosage of phage therapy eliminated 7 log colony-forming units (CFU)/g of fibrin clots in 6 h. Phage-resistant mutants formed again after 24 h, which was prevented by administering ciprofloxacin with the phage. Single-dose phage therapy successfully treated 64% of rats *in vivo* and destroyed 2.5 log CFU/g vegetation in 6 h.	Mortality	None	Oechslin et al. (69) France	
	Mice (BALB/c)	Intraperitoneal	*A. baumannii*	Endocarditis: A single dosage of phage therapy eliminated 7 log colony forming units (CFU)/g of fibrin clots in 6 h; mutant strains resistant to the phage proliferated once more after 24 h but were eradicated by administering ciprofloxacin in combination. Single-dose phage therapy successfully treated 64% of rats *in vivo* and destroyed 2.5 log CFU/g vegetation in 6 h.	Mortality	None	Patel Shesh R et al. (70) India
	Mice (BALB/c)	Intraperitoneal	*K. pneumoniae*	A single dose of the phage cocktail with 10^5^ PFU/mouse protected the mice from fatal consequences at any stage of septicaemia. However, a higher phage dose of 10^12^ PFU/mouse was lethal in the early hours of septicaemia, while this high dose is non-fatal in the later stages.	Mortality	None	Singh et al. (71) India
Gastrointestinal tract Infections	Mice (BALB/c)	Oral	*E. coli*	In a dose-dependent way, mixing the cocktail with water to consume for 24 h significantly decreased ileal *E. coli* concentrations and only slightly decreased fecal *E. coli* concentrations.	Bacterial load	None	Maura et al. (72) France	
	Rabbit (Outbred New Zealand White rabbits)	Oral	*Vibrio cholerae*	In contrast to the treatment given before a bacterial challenge, administering the phage cocktail orally after one considerably reduced the number of bacteria excreted.	Bacterial load	None	Jaiswal et al. (73) India	
	Mouse (Swiss Albino)	Oral	*Vibrio cholerae*	Mice treated with a phage cocktail reduced the number of colonies per gram by 3 logs. However, mice treated with ciprofloxacin reduced viable counts to 5 logs/g of tissue homogenates. While the oral rehydration solution failed to reduce the number of viable bacteria, disease progression was much slower.	Mortality and Bacterial load	None	Jaiswal et al. (74)India	
	Mice (BALB/c)	Oral and Intraperitoneal	*V. parahaemolyticus*	Mice that received phage treatment an hour after receiving i.p. bacteria (MOI of 0.1, 1, and 10) were shielded against illness and mortality.	Mortality	None	Jun et al. (75) South Korea	
	Hamster (Syrian golden)	Oral	*Clostridium difficile*	Compared to untreated animals, phage treatment delayed the onset of symptoms by 33 h and reduced *C. difficile* colonization 36 h after infection.	Mortality	None	Nale et al. (76) United Kingdom	
	Mice (BALB/c)	Drinking water	*E. coli*	The levels of *E. coli* were dramatically reduced throughout the gut after a single dosage of a combination of the three bacteriophages, with far less disruption of the microbiota diversity than antibiotics.	Bacterial load	None	Galtier al (77). France	
	Mice (BALB/c)	Oral	*Salmonella enteritidis*	After ten days of treatment, oral administration of phage (10^9^ PFU/mL) protected mice from salmonellosis and prevented weight loss.	Mortality and Bacterial load	None	Nikkhahi et al. (78) Iran	
	Mice (BALB/c)	Oral	*E. coli*	Over the ten-day study, mice were protected from enteropathogenic *E. coli* by a single dose of phage (2 × 10^9^ PFU/mL).	Mortality	None	Vahedi et al. (79) Iran	
	Mice (Swiss albino)	Intraperitoneal and Oral (Drinking water)	*S. typhimurium*	In Swiss albino mice, *S. typhimurium* acute infection and chronic carrier were established. However, when mice received phage (10^5^ PFU/mL) intraperitoneally at 24-h intervals, the severity of the acute disease was lessened, and they were back to normal after nine days. While a high count (10^12^ PFU/mL) phage cocktail given orally within seven days of feeding completely cured the carrier condition.	Bacterial load	None	Yadav et al. (80) India
	Mice (BALB/c)	Oral or enteral	*Salmonella enterica*	Animals infected with *S. enteritidis* were treated with a single dose of phage SE20 (2 × 10^8^ PFU/mL), which caused the animals to develop hepatomegaly and splenomegaly but not gastrointestinal problems.	Bacterial load	None	Dallal et al. (81) Iran
	Mice (ICR)	Intraperitoneal	*V. vulnificus*	All phage-treated mice (MOI = 10) died within 48 h, while the survival rate of phage-treated mice (MOI = 100 and 1,000) was 50 and 70%, respectively, after seven days. Nevertheless, untreated mice died in 12 h.	Mortality and bacterial load	None	Kim et al. (82), South Korea
Respiratory tract infections (Upper respiratory tract infections)	Mice (BALB/c)	Intranasal	*S. aureus*	By day seven after treatment, a single phage (MOI 1 or 10) had completely eradicated *S. aureus* from the nares compared to the control group. Mupirocin treatment yielded a comparable result, whereas phage treated with mupirocin exhibited 100% clearance by day 5.	Bacterial load	None	Chhibber et al. (83) India
	Sheep (Marine-cross)	Intranasal	*S. aureus*	It was discovered that a phage cocktail (10^6^ PFU/mL) was used for flushing frontal sinusitis once daily for three days in decolonized nostrils and was safe for short-term use. Treatment with EDTA has a comparable result. Phage and EDTA, however, worked better together.	Bacterial load	None	Drilling et al. (84) Australia
	Sheep	Intranasal (Extension canula)	*S. aureus*	Application of phage to the frontal sinuses of sheep was safe for 20 days; the paranasal sinus mucosa did not experience any inflammatory infiltration or tissue damage.	Bacterial load	None	Drilling et al. (85) Australia
	Sheep (Merine cross wethers)	Intranasal	*P. aeruginosa*	In sheep frontal sinuses, a 7-day-old biofilm was dramatically decreased by a phage cocktail (10^8^ to 10^10^ PFU/mL) applied twice daily with no safety issues noted.	Bacterial load	None	Fong et al. (2019) (86) Australia
Lower respiratory tract infections	Mice (BALB/c)	Intranasal	*P. aeruginosa*	A single dose of phage (10^8^ PFU/mL) administered to immunocompetent mice 2 h after the onset of infection (curative treatment) enabled a survival rate of over 95%. On the other hand, a 4-day preventive medication (a single dose) led to a 100% survival rate.	Mortality	None	Morello et al. (87) France	
	Mice (BALB/c)	Intranasal	*P. aeruginosa*	Phage cocktail reduced biofilms in cystic fibrosis bronchial epithelial CFBE41o cells. Tests on infected mice with a lux-tagged strain showed a 4-log reduction in the lungs after 6 h of treatment.	Mortality	None	Alemayehu et al. (88) Ireland	
	Mice (BALB/c)	Intranasal vs. Intraperitoneal	*Burkholderia cenocepacia*	The analysis of several phage delivery techniques. Compared to mice receiving intraperitoneal injections of phage therapy, those receiving aerosolized phage therapy significantly reduced bacterial load.	Bacterial load	None	Semler et al. (27) Canada	
	Mice (Swiss-webster)	Intranasal	*K. pneumoniae*	Mice were protected from lethal pneumonia by an intranasal injection of 2× 10^9^ PFU/mouse two hours after *K. pneumoniae* inoculation. The phage-treated mice had a lower lung bacterial burden than the untreated control group. In addition, reduced weight loss and inflammatory cytokines in their lungs were observed.	Mortality	None	Cao, Fang et al. (89) China	
	Mice (BALB/c)	Intraperitoneal	*K. pneumoniae*	Even when therapy was initiated three days after the development of pneumonia, liposome-encapsulated phage (LP) was effective in curing infection. On the other hand, non-liposomal phage offered protection when given 24 h after infection.	Mortality	None	Singla et al. (90) India	
	Mice (BALB/c)	Intravenous	*S. aureus*	Similar survival rates were seen in mice treated after 72 h of infection with a single dosage of clindamycin (8 mg/kg/body weight), 10^8^ PFU/mL phage, or combination therapy (clindamycin plus phage). However, the mice in the phage control group were more active than those in the clindamycin, phage, and combination therapy groups.	Mortality and bacterial load	None	Oduor et al. (91) Kenya	
	Mice (BALB/c)	Intranasal	*P. aeruginosa*	Phage administered at an MOI of 10 could eliminate the lungs of infection 24–48 h after infection. Phage exposed to UV light had no protective effect, however.	Mortality and bacterial load	None	Waters et al. (92) United Kingdom	
	Sheep	Intranasal (Extension canula)	*S. aureus*	NOVO12 (a two-phage cocktail) was applied topically to the frontal sinus for 20 days, and this treatment was considered safe because it did not cause any tissue damage or inflammatory reactions.	Mortality	None	Drilling et al. (93) Australia	
	Mice (Swiss mouse)	Endotracheal route	*P. aeruginosa*	The bacterial load in the lungs was decreased by 5.3 logs compared to the untreated group when phage dry powder formulation (2 mg/mice) at a dosage of 2 × 10^7^ PFU/mg was applied two hours after a bacterial challenge.	Bacterial load	None	Chang et al. (94) Australia	
	Mice (C57BL/6)	Intraperitoneal	*P. aeruginosa*	When administered 4 h after infection, single phage treatments (10^9^ PFU/mL) significantly reduced lung infection in mice (>4 logs) and shielded them (>65%) from a lethal disease. At the same time, animals in the untreated groups all died by day three post-infection.	Mortality	None	Jeon et al. (95) Republic of Korea	
	Mice (BALB/c)	Intravenous	*E. coli*	Antibiotics and bacteriophages had comparable outcomes, but bacteriophages were more effective at reducing bacterial burden. Additionally, bacteriophage therapy did not promote excessive inflammation but tended to reduce it and corrected blood cell count irregularities more quickly than antibiotics.	Mortality and Bacterial load	None	Dufour et al. (96) France	
	Mice (BALB/c)	Intraperitoneal	*K. pneumoniae*	After a 10-min bacterial challenge, the mice were administered phage (1.75× 10^8^ PFU/animals), which ensured 100% survival and no signs of infection. This prevented all of the mice from death. This therapeutic effect was not observed when the phage suspension was administered an hour after the bacterial challenge. In this instance, the survival rate was still 100% after 24 h, but the mice’s general health significantly declined, and 48 h after infection, their survival rate decreased to 12.5% (1/8 mice). No rescue effect was observed when a phage was given three hours after a bacterial challenge.	Mortality and bacterial load	None	Horváth et al. (97), Hungary
	Mice (BALB/c)	Intraperitoneal and oral	*K. pneumoniae*	The phage Kp_Pokalde_002 was administered intraperitoneally (IP) and orally to the infected mice at an MOI of 1.0 (~1 × 10^7^ PFU/mouse). Phage treatment was used to treat sick mice by oral and intravenous methods. As a result, the bacterial burden in the blood and lungs decreased significantly (3-7 log10 CFU/mL) in the treatment group.	Mortality and bacterial load	None	Dhungana et al. (98) Nepal
	Rats (Wistar)	Respiratory and intravenous	*S. aureus*	The animals received aerophages, intravenous (IV) phages, IV linezolid, IV and aerophages, and a combination of IV linezolid and aerophages. Aerophage therapy considerably increased survival (by 50%). In addition, Aerophages and IV phage therapy significantly improved survival compared to either treatment alone, even though IV phage therapy alone only led to a 50% survival rate. Finally, aerophages were added, but no synergistic impact was seen.	Mortality and bacterial load	None	Prazek et al. (99), Switzerland
	Mice (Wild-type C57BL/6 J)	Intraperitoneal	*K. pneumoniae*	Phages were injected intraperitoneally (IP) at various times (1, 8 and 24 h after bacterial infection) and at various MOIs (an MOI of 1 or 10). The treatment duration, not the phage dose, significantly impacted survival. A control group of mice that received saline treatment developed a severe illness quickly in all of them. However, mice treated with viable phages survived those effectively receiving saline.	Mortality and bacterial load	None	Hesse et al. (100), United States
Skin and soft tissue infections	Mice (BALB/c)	Intraperitoneal	*K. pneumoniae*	When administered immediately after a bacterial challenge, the Kpn5 phage efficiently treated mice with a burn wound infection and could save about 26.66% of the mice even after 18 h of bacterial challenge. In addition, pro-inflammatory cytokines (IL-1beta and TNF-alpha) and anti-inflammatory cytokines (IL-10) were also found in much-reduced concentrations in the serum and lungs of mice treated with phage.	Mortality	None	Kumari et al. (101) India	
	Mice (BALB/c)	Topical	*K. pneumoniae*	Phage Kpn5 mixed with hydrogel applied topically at an MOI of 200 on the burn wound site could prevent infection in mice compared to multiple silver nitrate and gentamicin treatments.	Mortality	None	Kumari et al. (102) India	
	Pig (Yorkshire)	Topical	*S. aureus, P. aeruginosa and A. baumannii*	The pig wound infection model used a phage cocktail of six different phages. Results varied depending on the species, but mechanical debridement generally had a positive effect. Compared to the pig model, the rodent model demonstrated more significant improvement.	Mortality	None	Mendes et al. (103) Portugal	
	Mice (BALB/c)	Topical	*S. aureus*	The efficacy of the phage MR-10, which was locally applied at a dose of 10^8^ PFU/mL (MOI-100), was equivalent to that of the antibiotic linezolid. However, combination therapy using bacteriophage and linezolid was more successful in preventing the infection process than antibiotics or phage used alone.	Bacterial load	None	Chhibber et al. (104) India	
	Mice (BALB/c)	Sub cutaneous	*Mycobacterium ulcerans*	33 days after the bacterial challenge, a single subcutaneous injection of mycobacteriophage D29 reduced pathology and prevented the development of ulcers. This protection significantly reduced the number of *M. ulcerans*, increased levels of cytokines (including IFN-), and generated a lymphocytic/macrophage-profiled cellular infiltration.	Bacterial load	None	Trigo et al. (105) Portugal	
	Mice (BALB/c)	Intraperitoneal	*E. coli*	Bacteriophage (T4) was administered intraperitoneally after being modified by phage display to contain anticancer Tyr-Ile-Gly-Ser-Arg (YIGSR) peptides. In mice receiving YIGSR-displaying phage treatment, tumor growth was slowed. In addition, mice receiving phage treatment had considerably lower wound severities, bacterial loads, and inflammatory markers.	Bacterial load	None	Dabrowska et al. (106) Poland	
	Rats (Wistar)	Topical	*A. baumannii*	Comparing the phage-exposed group to the antibiotic-treated uncontrolled diabetic rats and the control group, a substantial decrease in infection, epithelialization time, and wound contraction was seen. Furthermore, the rats, including the phage controls, showed no signs of generalized sepsis and seemed remarkably healthy.	Bacterial load	None	Shivaswamy et al. (107) India	
	Mice (Swiss albino)	Injection	*P. aeruginosa*	The number of bacterial cells was significantly reduced. The number of phages was significantly increased when an 11-phage cocktail was added to subcutaneous bags containing two catheter sections loaded with biofilm.	Bacterial load	None	Basu et al. (108) India	
	Mice (BALB/c)	Subcutaneous and topical	*A. baumannii*	Treatment with the phage Abp1 (5.0 × 10^8^ PFU/mL) had significant therapeutic effects in mice models with local and systemic *A. baumannii* infection. Compared to mice who received either systemically administered phage or no treatment, mice that received locally applied phage had much-reduced wound sizes.	Mortality	None	Yin et al. (67) China	
	Rat (Wistar)	Intramuscular	*S. aureus*	Rats treated with the transfersome-entrapped phage mixture recovered from the experimental thigh infections in 7 days compared to the 20 days required for untreated animals. Even when given 12 h after infection, the transfersome-entrapped phage cocktail protected all test animals (without fatalities).	Mortality	None	Chhibber et al. (31) India	
	Mice (BALB/c)	Topical	*K. pneumoniae*	A phage cocktail comprising five and one individual phage was compared for their effects. Despite the successful results of single-phage therapy, the phage cocktail demonstrated superior outcomes and significantly delayed the emergence of resistant mutants.	Bacterial load and wound size	None	Chadha et al. (109) India	
	Mice (BALB/c)	Intraperitoneal	*K. pneumoniae*	The efficacy of a liposome-encapsulated phage cocktail against a free phage cocktail in treating a mouse burn wound infection was compared. The animals treated with a liposomal entrapped phage cocktail had a significantly lower bacterial load in the blood and major organs.	Mortality and Bacterial load	None	Chadha et al. (110) India
	Mice (BALB/c)	Topical	*S. aureus*	A single injection of a 10 MOI bacteriophage proved very successful in treating diabetic mice. Surprisingly, linezolid was ineffective in diabetic mouse models. Concomitant therapy (phages + linezolid) did not show any antibacterial synergy. A single injection of the phage had the same impact on non-diabetic control animals as linezolid had on non-diabetic infected animals.	Bacterial load	None	Albac et al. (111) France
	Mice (Swiss)	Topical or superficial	*S. aureus*	Phage therapy enhanced clinical recovery and decreased local bacterial load 7 and 14 days after infection. Unlike antibiotics, phage therapy did not cause the gut microbiota of the treated animals to become less diverse. The mouse microbiota’s alpha and beta diversity was reduced by amoxicillin. Additionally, while phage treatment did not affect the microbiota, it disrupted architecture even seven days after treatment ended.	Mortality and bacterial load	None	Huon et al. (112), France
	Mice (BALB/c)	Subcutaneous	*S. aureus*	Linezolid and subcutaneous phage injections were administered after the mouse air pouch model was established. The phage MR-5 dramatically decreased bacterial load (extracellular and intracellular), both alone and in combination with linezolid (showing synergy), accelerating the clearance of pouch infection.	Mortality and bacterial load	None	Kaur et al. (113) India
	Mice (BALB/c)	Topical	*S. aureus*	A single phage (a combination of three phages, J-Sa36, Sa83, and Sa87) reduced the bacterial burden with an efficacy comparable to or greater than vancomycin treatment. On the other hand, wounds from mice given saline treatment did not heal and grew larger, more infected, ulcerated, and suppurative.	Bacterial load	None	Kifelew et al. (114), Australia
Eye and ear infections	Dog	Topical	*P. aeruginosa*	A single phage cocktail (10^5^ PFU/ear) injected directly into the auditory canal after 48 h significantly decreased clinical scores without exhibiting any evidence of inflammation or additional adverse effects.	Bacterial load	None	Hawkins et al. (115), United Kingdom
	Mice (C57BL/6)	Topical	*P. aeruginosa*	Phage KPP12 eye drops administered as a single dose significantly accelerated the healing process while maintaining the structural stability and transparency of the infected cornea. The treatment with KPP12 also reduced neutrophil infiltration and significantly enhanced bacterial clearance in the infected cornea.	Bacterial load	None	Fukuda et al. (116) Japan
	Mice (C57BL/6)	Topical	*P. aeruginosa*	In the animal model for keratitis, a cocktail of two phages reduced bacteria. The keratitis caused by *P. aeruginosa* could be entirely prevented by the phages. Additionally, phages may prevent equine keratitis better than the current preventative use of antibiotics.	Bacterial load	None	Furusawa et al. (117) Japan
Other infections	Rat (Wistar)	Intramuscular	*E. faecalis*	When used at 37°C, phage survival was not impeded by formulation in poloxamer P407 media. This continued to be evident for a month. After formulation with poloxamer P407, the phage cocktail showed antibacterial activity and eradicated planktonic *E. faecalis* after 1, 2, 8, 14, 21, and 28 days.	Bacterial load	None	Schlezinger et al. (118) Israel
	Mice (SD mice)	Intraperitoneal	*A. baumannii*	Phage (5.0 × 10^8^ PFU) in PBS was administered intraperitoneally right after infection. Phages were reinjected after 12 h. In the control group, on the first day after infection, the infected mouse began to die, and within a week, all 12 died. In the phage treatment group, the sick mouse died three days later, and eight mice were still alive one week later.	Mortality	None	Jiang et al. (119) China
	*Galleria mellonella*	Haemolymph	*A. baumannii*	This study showed that combining the phage (MOI = 1) and meropenem, which increased larval survival from 35 to 77%, resulted in the greatest prolongation of *G. mellonella* larval survival.	Mortality	None	Grygorcewicz et al. (120), Poland

## Bacteriophage therapy in clinical infections

While phage therapy used with compassion (a profound awareness and empathy for another’s suffering) has recently increased with numerous case reports claimed to have cured patients, clinical trials intended to demonstrate its efficacy per current regulatory requirements have officially failed. Given the issue of rising antibiotic resistance, there needs to be an important decision on the role of phage therapy in contemporary medicine. The dramatic surge in case reports of treated patients reflects the growing interest in phage therapy. Additionally, various journals (Front Microbiol., Front Pharmacol., Viruses, Antibiotics, Pathogens, Microorganisms, etc.) have recently published special issues and study subjects on phage therapy (121).

Since their discovery, phages have been widely used in Eastern Europe and the former Soviet Union; as a result, their medical systems now incorporate the therapeutic use of phages. However, a thorough scientific analysis of this potential therapy has recently been conducted (41, 122). Abedon offered a list of essential requirements that should be carefully considered and reported in phage therapy (123). The effectiveness of clinical research depends on the proper characterization and selection of the phages, the participants (humans), and the target microorganisms. Though necessary, additional information like formulations, dosages, and potencies are only helpful when used with clearly defined and well-planned goals. The quality of future studies would increase with more thorough reporting, enabling the replication and expansion of earlier investigations. Choosing suitable disease targets for phage therapy is another factor to consider. Phage mixtures provide a wide range of activity when contemplating monotherapy or combination therapy techniques and lessen the possibility of resistance development. Additionally, it significantly increases the difficulty of determining how each phage in a cocktail would affect inflammation, the possibility of gene transfer, and the emergence of phage resistance (124).

In several world regions, bacteriophage therapy has been utilized for many years to treat bacterial infections. As reviewed by Uyttebroek et al. (125), Kutter et al. (41) Abedo et al. (35) and described in Marza et al. (126), encouraging results have been documented. Although bacteriophage therapy has been used for many years, there are few clinical studies in this field, raising many doubts about its effectiveness against infectious diseases. However, the growing demand for novel antimicrobial therapies drives the development of bacteriophage therapeutics for diverse diseases. These require the completion of extensive clinical trials per United States FDA or European EMA requirements (127). The phage’s bactericidal activity, and concerns about potential toxic shock must also be addressed.

### Musculoskeletal infections

Fish et al. (128) presented a continuous case series examining the effectiveness of treating infected and poorly vascularized toe ulcers with the exposed bone when the recommended antibiotic therapy with topically applied *S. aureus*-specific phages failed. After providing standard wound care, the phage solution was topically given to the ulcerations weekly. All infections responded to the phage therapies, and infected bone debridement resulted in an average healing time of 7 weeks for the ulcers. In order to retain hallux function and treat an ulcer with extremely poor vascularity, 18 weeks of therapy were required.

The case report by Fish et al. (129) successfully treated distal phalangeal osteomyelitis in a 63-year-old diabetic woman with long-term bacteriophage follow-up. In a different study, Fish et al. (130) reported using the commercial formulation of the extensively researched anti-*Staphylococcal* bacteriophage Sb-1 from Eliava Bio Preparations to cure diabetic toe ulcers successfully. In difficult-to-close toe ulcers containing contaminated/infected bone, topical administration of a single *S. aureus*-targeting bacteriophage proved successful.

According to a study by Ferry et al. (131) local injections of a bacteriophage mixture during debridement, antibiotics, and implant retention (DAIR) procedures to treat recurrent *S. aureus* chronic joint prosthesis infection (PJI) were effective, safe, and associated with clinical success.

Cano et al. (132) reported intravenous injections of a single phage (KpJH462) to a 62-year-old diabetic patient with prosthetic infection due to *K. pneumoniae* complex, resolving local symptoms and signs of infection and restoring function. In addition, a trend toward reduction in biofilm biomass was observed 22 h after exposure to KpJH462.

A case study of a 72-year-old man with a chronic methicillin-resistant *S. aureus* prosthesis infection was published in 1971 by Doub et al. (133) Bacteriophage therapy was stopped following the third intravenous dose due to a rare, reversible transaminitis. Despite this, the patient’s severe chronic infection was successfully treated and eradicated.

A persistent methicillin-sensitive *S. aureus* (MSSA) prosthetic knee joint infection in a 61-year-old woman was successfully treated after a second cycle of bacteriophage therapy given during a two-stage replacement procedure, according to Ramirez-Sanchez et al. (134) The study also highlighted the success of bacteriophage therapy with a single lytic phage, the safety and effectiveness of intravenous and intra-articular infusions, and the development of serum neutralization with continued treatment.

The study by Onsea et al. (135) reported the successful use of bacteriophage therapy for patients with severe musculoskeletal infections. There was no recurrence of infection with the causal strains following a single phage therapy with concurrent antibiotics, with follow-up periods ranging from 8 to 16 months. In addition, the phage application protocol has not been associated with harmful side effects.

In a case report, Chan et al. (136) described the therapeutic application of phage OMKO1 to a chronic *P. aeruginosa* infection of an aortic Dacron graft with a related aortocutaneous fistula. Phage OMKO1 and ceftazidime were administered only once, and the infection seemed to clear up with no signs of recurrence.

### Urinary tract infections

Ujmajuridze et al. (137) conducted a prospective cohort study to examine the efficacy of prophylactic bladder instillation in patients undergoing transurethral prostate resection. Nine patients got postoperative phage therapy by bladder instillation after a preoperative examination of asymptomatic individuals for the presence of uropathogens in midstream urine. No adverse events were reported. In six cases, the bacterial burden was also decreased. The outcomes of this two-phase, prospective trial indicated that tailored bacteriophage therapy for treating UTI might be efficient and secure.

A Dutch case study by Kuipers et al. (138) showed how bacteriophage was utilized successfully to treat chronic recurring urinary tract infections in a kidney transplant recipient caused by extended-spectrum lactamase (ESBL)-positive *K. pneumoniae*.

The study by Corbellino et al. (139) on long-term, multisite colonization by an MDR *K. pneumoniae* strain in a patient with a single kidney, cutaneous ureterostomy, and permanent ureteral stent resolved after 3 weeks of treatment with personalized BT given both orally and intrarectally.

In refractory *P. aeruginosa* urinary tract infection accompanied by bilateral ureteric stents and bladder ulcers, Khawaldeh et al. (140) demonstrated the benefit of adjuvant bacteriophage therapy after recurrent failure of antibiotics alone. The dynamics of bacteria and bacteriophages in urine point to a self-perpetuating and self-limiting infection, and no bacteriophage-resistant bacteria formed.

In a clinical trial by Quin et al. (141), phage therapy was utilized to treat a man with multiple urinary tract infections caused by MDR *K. pneumoniae*. However, three phage therapies were unsuccessful due to polyclonal co-infectious cells in his renal pelvis and bladder. Therefore, a percutaneous nephrostomy was done on the patient (PCN) following analysis. In addition to receiving antibiotic therapy, a mixture of bacteriophages chosen for their ability to attack all 21 diverse isolates were simultaneously irrigated via the kidney and bladder. The patient eventually made a full recovery with an improved bladder.

Other studies investigating the use of bacteriophage therapy in individuals with persistent urinary tract infections have shown its efficacy in overall clinical improvement (symptom alleviation and prevention of UTI recurrence) and bacterial eradication (142–144).

### Skin and soft tissue infections

Equal healing rates were observed in the control and test groups of Rhodes D. et al.’s phase I clinical trial on bacteriophage therapy for treating human venous leg ulcers. In the study, 39 patients had their chronic blood vessel leg ulcers treated for 12 weeks with either a bacteriophage product or a placebo. No adverse events or safety issues were recorded (145).

The PhagoBurn Section 1/2 study by Jault P. et al. (5) was another clinical trial that compared standard care (Sulfa-drug silver emulsion cream) with a cocktail of twelve anti-*P. aeruginosa* lytic phages applied via an alginate dressing for a seven-day treatment of burn wound infection in 25 patients. Unfortunately, bacteriophage therapy has shown to be ineffective due to several factors, including variations in the maximum bacterial load between treatment groups and a significantly lower-than-expected bacteriophage dosage due to a concentration decrease during manufacturing and the fall in titres (during storage) were not checked before using them in the clinical trial.

A study by Patel et al. (146) using a customized bacteriophage preparation in managing chronic nonhealing wounds reported 100% microbiological eradication after 3 months with a healing rate of 81.2%. Additionally, the study suggested that specific phage therapy is equally effective whether a patient has diabetes or not, despite healing being slower in the former cluster. Another study by Gupta P et al. (147) examined the impact of topical bacteriophage therapy on chronic nonhealing wounds infected with *E. coli*, *S. aureus*, and *P. aeruginosa*. They found that after 3 to 5 doses, there was a significant improvement in wound healing with no signs of infection, both clinically and microbiologically.

Both studies offered nearly unambiguous proof that topical phage therapy helped patients who had not responded to conventional therapy completely heal their wounds. Furthermore, the isolates from the research population’s chronic wounds showed significant resistance to the most widely used antibiotics. Neither study’s participants experienced adverse effects, tissue breakdown, or recurrent infections during or after therapy.

In order to determine the impact of bacteriophage on the healing process, Bhartiya et al. (148) conducted a non-randomized, prospective, open-label, blinded, case–control study on infected acute traumatic wounds. The results of this investigation are promising. In patients receiving phage therapy, the average time needed to achieve sterility, complete wound granulation, and primarily intended healing was half that of the control group. The financial analysis also favored bacteriophage therapy (BT), as only 1/3 of the costs were incurred in the BT group compared to the control group.

Marza et al. (126) reported clinical improvement and bacterial elimination after phage therapy in a patient with an infected burn wound. In contrast, Rose T. et al. (149) discovered that phage therapy had no therapeutic effects on burn wounds. Bacteriophage use, however, had no adverse effects.

### Ophthalmic infections

Infectious keratitis induced by Vancomycin-resistant *S. aureus* was described in the study by Fadlallah et al. (150) Phage treatment was given intravenously and topically in the form of eye drops and nasal spray. There were no concurrent antibiotics given. The infection was effectively treated, as evidenced by stabilizing ocular symptoms and observing negative cultures. The six-month follow-up period had showed no adverse effects and no reinfection.

### Intensive care for patients with sepsis

Many studies have not been done on patients who received phage therapy for severe sepsis. *S. aureus*, *P. aeruginosa*, *E. coli*, *K. pneumoniae*, *Proteus mirabilis*, *Morganella morganii*, and *Enterobacter* spp. were the pathogens that were frequently isolated. Phage treatment was given intravenously, intramuscularly, or both intramuscularly and locally. Of the 109 patients, 85 (78%) received antibiotics simultaneously. Adverse incidents were not reported. Follow-up lasted anywhere between 20 days and 4 months. As a result, 90 (83%) of the 109 patients had improved vital signs, and two wholly eradicated their bacteria. Unfortunately, phage therapy proved ineffective in 20 patients; three died despite the initial improvement, and five died shortly after the treatment began (151–153).

### Gastrointestinal infections

A 68-year-old diabetic patient with necrotizing pancreatitis was described in a case report by Schooley et al. (154) with an MDR *A. baumannii* infection. The patient’s clinical course was reversed, the bacteria were cleared up, and they recovered after receiving these bacteriophages intravenously and through percutaneously into the abscess cavities.

### Cardiovascular infections

Three studies detail individuals with aortic graft and left ventricular assist system infections that were difficult to cure and were infected by *P. aeruginosa* or *S. aureus* (136, 155, 156). Two patients received local phage therapy, while one received intravenous phage therapy. Antibiotics were given concurrently to all patients. After receiving a local phage application, one patient reported nausea. There was a clinical improvement in all cases; one patient showed evidence of bacterial eradication. The follow-up period lasted 7-9 months.

### Respiratory infections

Several case studies have been published regarding phage therapy and patients with respiratory infections (157–166). Phage therapy was administered intravenously, orally, and intravenously using a nebulizer. Phage treatment was well tolerated in each case and had no adverse side effects. Furthermore, clinical improvement was observed in most cases, including general improvement and reduced sputum and coughing.

### Other infections

Nine patients with chronic rhinosinusitis caused by *S. aureus* were treated with intranasally given phage therapy in a prospective study by Ooi et al. (167). The treatment was examined for safety and preliminary efficacy. Phage therapy was well tolerated, and the side effects (such as diarrhea, epistaxis, signs of an upper respiratory infection, oropharyngeal discomfort, rhinitis, and a drop in serum bicarbonate) were modest. All nine patients saw improvements in their clinical conditions, and the bacterial load was reduced; however, only two (22%) of the nine patients had their bacteria wholly eradicated. There was a seven-day follow-up period (167).

Despite many positive case studies, there still needs to be robust scientific evidence from well-planned, monitored and regulated clinical trials supporting bacteriophage therapy. However, more recently, a rise in the number of bacteriophage therapy-related articles, books, and reviews and commercial bacteriophage companies focusing on a specific market suggests that the scientific communities and pharmaceutical companies are becoming more eager to integrate bacteriophage therapy into conventional medical practice (127).

Phage therapy may be helpful for bacterial illnesses that are challenging to cure. Additionally, this treatment is generally considered safe because it has a low incidence of side effects and is given via various administration routes. Even though phage therapy appears to be a promising strategy in the fight against untreatable infections and antimicrobial resistance, high-quality studies are desperately needed to advance our understanding of the long-term effects of this treatment. When taken orally and intravenously, getting more knowledge about the pharmacokinetics and pharmacodynamics of the bacteriophage (cocktails) is necessary. Additionally, endotoxin, bacterial, and viral contamination testing for bacteriophage solutions should be done (Table 4).

**Table 4:** Ongoing clinical trials of phage.

Name of the study	Phase	Registry date	Clinical trial registry number	Trial result	Public data
A phase 1b/2 trial of the safety and microbiological activity of bacteriophage therapy in Cystic fibrosis subjects colonized with *Pseudomonas aeruginosa*	I/II	12 July 2022	NCT05453578	–	Trial design (168)
Cystic Fibrosis bacteriophage study at Yale (CYPHY)	I/II	24 December 2020	NCT04684641	–	Trial design (169)
A randomized, placebo-controlled, double-blind clinical trial of therapeutic bacteriophage preparation in chronic antibiotic-resistant wound infections at Banaras Hindu University, Varanasi, India.	I/II	8 December 2021	CTRI/2021/12/038527	–	Trial design (170)
Phage Therapy for the treatment of urinary tract infection	I/II	13 September 2022	NCT05537519	–	Trial design (171)

### Worldwide phage development organizations

Phage therapy has been used for many years in certain countries, primarily in Eastern Europe, although it has yet to gain widespread acceptance in Western nations. This is partly due to the need for more commercial phage production, making it difficult to standardize and regulate phage therapy products.

However, several companies have recently been established to develop and commercialize phage-based products. These companies use various methods to produce phages, including isolating phages from environmental samples, genetically engineering phages, and producing phages using fermentation or other bioprocessing techniques. One of the main challenges in commercial phage production is ensuring consistent quality and purity. This is important to ensure that the phages are safe and effective for medical applications. In addition, companies use various methods to standardize and test their phage products, including use of bioassays to measure phage activity and testing for endotoxins and other contaminants. Several companies worldwide are developing and commercializing phage-based therapies for various bacterial infections. Here are some examples, summarized in Table 5, including the name of the company, its location, and the company website.

**Table 5:** Global distribution of companies exploiting phages for therapeutic purposes (7, 172–174).

Company	Locations	Web site address
Adaptive Phage Therapeutics	Maryland, United States	https://aphage.com/
AmpliPhi Bioscience Corporation	Virginia, United States	http://www.ampliphibio.com
Armata Pharmaceuticals, Inc	California, United States	https://www.armatapharma.com/
Aziya Immuno-preparat	Tashkent, Uzbekistan	https://aziyaimmunopreparat.uz/
BigDNA	Edinburgh, United Kingdom	http://www.bigdna.com/
Biopharm Ltd.	Tbilisi, Georgia	https://biopharm-ge.com/
Biophage Pharma Inc.	Montreal, Canada	http://www.biophage.com/
BiomX	United States	https://www.biomx.com/
CJ CheilJedang Corporation	Seoul, South Korea	https://www.cj.co.kr/
Elanco Food Solutions	Illinois, United States	https://www.elanco.com/
EBI Food safety	Wageningen, the Netherlands	https://www.ebifoodsafety.com/
Ellis Day Skin Sciences	California, United States	https://www.ellisdayskinscience.com/
Eliava Bio Preparations Ltd.	Tbilisi, Georgia	https://pha.ge/
Exponential Biotherapies, Inc.	Virginia, United States	http://www.expobio.com/
Gangagen Biotechnologies Pvt. Ltd.	Bangalore, India	https://gangagen.com/
Gangagen Inc.	California, United States	https://gangagen.com
Hexal Genentech	Holzkirchen, Germany	http://www.hexal.de/
Innophage	Porto, Portugal	http://www.innophage.com/
Intralytix	Maryland, United States	http://www.intralytix.com
Jafral Ltd.	Ljubljana, Slovenia	https://jafral.com/
JSC Biochimpharm	Tbilisi, Georgia	https://biochimpharm.ge/
Locus Biosciences	North Carolina, United States	https://www.locus-bio.com/
MB Pharma	Prague, Czech Republic	https://www.mbph.cz/?lang=en
MicroMir	Moscow, Russia	https://micromir.bio/eng
Microgen	Moscow, Russia	https://www.microgen.ru/en/
Novolytics	Coventry, United Kingdom	http://www.novolytics.co.uk/
New Horizons Diagnostics	Maryland, United States	http://www.nhdiag.com/index.htm
OmniLytics Inc.	Utah, United States	https://www.omnilytics.com/
Phage International, Inc.	California, United States	https://www.phageinternational.com/
Phage Biotech Ltd.	Rehovot, Israel	http://www.phage-biotech.com/
Phage Therapy Center	Tbilisi, Georgia	https://www.phagetherapycenter.com/
Phico Therapeutics	Cambridgeshire, United Kingdom	https://www.phicotx.co.uk/
Pherecydes Pharma	Ile-de-France, France	https://www.pherecydes-pharma.com/en/
Proclara Biosciences	Massachusetts, United States	http://www.proclarabio.com/
Phagelux	Sandy, Utah, United States	http://www.phageluxagrihealth.com/en/
Phagex	Kyiv, Ukraine	https://bacteriophages.info/en/
Special Phage Services Pty, Ltd.	New South Wales, Australia	http://www.specialphageservices.com.au/
SciPhage Biotechnology	Colombia, United States	https://sciphage.com/
Targanta Therapeutics	Massachusetts, United States	http://www.targanta.com/
Viridax™ Corporation	Florida, United States	http://www.dreamingrock.com/viridax/eviridax/

## FDA-approved bacteriophage-based products and regulatory challenges

Phage therapy treatments have only been carried out in Western medicine under the laws of the Helsinki Declaration, which was adopted by the 18th World Medical Association General Assembly (Helsinki, Finland, June 1964) as unproven interventions in clinical practice or out of compassion and with the patient’s informed consent (175). A new legal framework has just been installed in Belgium that permits phages to be used as an active pharmaceutical ingredient in a magistral preparation as long as specific logical requirements are met. A medicinal preparation is described as “any medicinal product prepared in a pharmacy following a medical prescription for an individual patient,” in line with Article 3 of Directive 2001/83 of the European Parliament and Article 6 quarter 3 of the Belgian medicinal law of 25 March 1964 (176, 177). The preparation must be explicitly made by a pharmacist from the various components per current pharmaceutical standards and under a Medical Doctor’s (MD) prescription for a selected patient (177). In 2006, the United States Food and Drug Administration (FDA) approved using a phage as an antibacterial (preventive) agent in “the ready-to-eat food,” The process of developing and marketing phage therapy medical products (PTMPs) can theoretically be completed while adhering to all regulations, but it is exceedingly expensive and time-consuming. Furthermore, given the ongoing evolutionary dynamics between bacteria and phages, it is possible that the phage products that are eventually launched on the market are already obsolete or will do so shortly due to the lengthy development times concerning the divergent evolution of the targeted bacterial populations (178).

The current approach for producing and marketing pharmaceutical products was primarily designed for static chemical drugs like antibiotics and typically includes the following elements: Producing using good manufacturing practices (GMP), Preclinical research, including *in vitro* and animal pharmacokinetic, pharmacodynamic and toxicological investigations\Phase I to IV clinical trials and centralized marketing authorization (177).

The PhagoBurn study showed that producing PTMPs under the established pathway is very expensive, time-consuming, and only sometimes leads to high-quality and effective products. The licensed GMP research product’s titer was reduced drastically (1000-fold) 15 days after manufacturing, and manufacturing and licensing consumed the majority of the time and money allotted to the study (5). Phage therapy is a form of “experimental treatment” legal in Poland. The modified Act of 5 December 1996 on the Medical Profession, issued in the Polish Law Gazette, 2011, No. 277 item 1,634, and Article 37 of the Declaration of Helsinki, serve as the foundation for this framework. Phage treatment is practically feasible in Poland under specific circumstances, such as informed consent, a doctor’s application, and a bioethics panel’s approval, but only without any other potentially viable and established treatment alternative (177). Under the ‘compassionate use’ regulation, phage therapy may occasionally be used in various nations. Australia and France are examples (140, 179). Despite the European Medicines Agency (EMA)’s advice, each nation appears to employ a unique approach to this treatment.

The U.S. Food and Drug Administration (FDA) has been attempting to create a legal framework for employing bacteriophages as a therapeutic modality. In the United States, phage therapy has been considered an investigational new drug (IND) and regulated under the FDA’s IND program. This program allows for the clinical testing of new drugs and biologics in humans before they are approved for use in the general population. Recently, the FDA has taken steps to establish a more formal regulatory framework for phage therapy. In 2019, the agency released a draft guidance document on developing bacteriophage products for treating bacterial infections. The guidance document provides recommendations for developing and submitting data to support the safety and efficacy of phage products (180).

The FDA’s draft guidance recommends that phage products be evaluated in well-controlled clinical trials designed to demonstrate safety and efficacy. The guidance also recommends manufacturing phage products using appropriate quality control measures to ensure consistency and purity. It can be an essential step in regulating phage therapy. It provides a roadmap for companies developing phage products and clinicians interested in using phage therapy to treat bacterial infections. The guidance also provides a basis for discussion between the FDA and industry stakeholders on developing safe and effective phage products (181). Phage products have also been successfully produced globally in various applications, supporting their safety and efficacy after the approval of the FDA and other regulating bodies. Companies’ names, product details, agency approval, and related applications of phages are compiled in Tables 6, 7.

**Table 6:** List of regulatory body-approved phage-based products for food-borne pathogens in food.

Product and company	Regulatory approbation	Applications	References
ListShield, Intralytix, Inc. (United States)	US FDA (2006) for direct application onto foods	Salami, sausage, shellfish (Ready-to-eat food), and food contact surfaces and environments:	(182)
EcoShield, Intralytix, Inc. (United States)	FDA (2011) cleared as “Food Contact Notification”. (Safe and suitable antimicrobial)	Red meat parts and trim intended to be ground	(183)
SalmoFresh, Intralytix, Inc. (United States)	FDA listed suitable in the production of organic foods	Poultry, fish, and shellfish, freshly processed fruits and vegetables	(184)
LISTEX Micreos EBI, Food Safety, (Netherlands)	FDA-Approved	Ready to eat meat, fish, and cheese	(185)
Agriphage, Omnilytics, United States	Environmental Protection Agency (EPA 2005) for use in agriculture	In agriculture, fruits, and vegetables	(186)
Compyshield™, Intralytix, Inc. (United States)	FDA Approved	Food additive for raw red meat	(187)
Shiga Shield™, Intralytix, Inc. (United States)	FDA Approved	*Shigella* removal from meat and vegetables	(188, 189)
EnkoPhagum, Brimorose Technology Corporation (United States)	FDA Approved	Removal of *Salmonella, Shigella, E. coli,* and *Staphylococcus* in meat products	(188)
SalmoPro™, Phagelux (China)	FDA Approved	As an antibacterial processing aid in food	(190)
Secure Shield E1, FINK TEC GmbH (Germany)	FDA Approved	Used in beef products, turkey, and other foods.	(191)

**Table 7:** List of commercial phage products for humans.

Product and company	Regulatory approval	Route of administration	Application	References
PhagUTI, Pherecydes, (France)	Phase I/II	Undefined	Treating *E. coli* Urinary tract infections (UTI)	(192)
EcoActive, Intralytix (United States)	FDA-approved IND, Phase 1/2a	Oral	Targeting adherent-invasive *E. coli*	(193)
AP-PA02; AP-PA03, Armata (United States)	FDA-approved IND, Phase 1b/2	Inhalation	Treatment of *P. aeruginosa*-related respiratory tract infections, particularly in Cystic Fibrosis patients.	(158)
PGX0100, Phagelux (China)	FDA-approved IND, preclinical	Transdermal	Spray and gel for burn care	(194)
Staphylococcal bacteriophage, Microgen (Russia)	Russian Federation national standard certification	Inhalation	Treatment of *Staphylococcal* Intestinal Disorders and Suppurative Inflammation	(195)
AP-SA01; AP-SA02, Armata (United States)	FDA-approved IND, Phase 1b/2	Intravenous	Diabetic foot ulcer treatment and management of resistant and refractory *S. aureus* bacteremia	(114, 158)
*Staphylococcal* Bacteriophage, Eliava Bio Preparation (Georgia)	Georgian Approval	Oral or intrarectal	Preventing and treating *Staphylococcal* infections and postoperative wound infections	(196)
BX003, BiomX (United States)	Phase I	Oral	The treatment aims at gut-residing *K. pneumoniae* bacterial strains in patients with primary sclerosing cholangitis (PSC) and Inflammatory bowel disease (IBD).	(197)
ShigActive™, Intralytix (United States)	FDA-approved IND,2021	Oral	Prevention of human diseases caused by *Shigella* infection	(198)
*Streptococcal* bacteriophage, Microgen (Russia)	Russian Federation national standard certification	Oral, topical, and intrarectal	Treatment diseases caused by *Streptococcus* spp.	(195)
Phagyo®spray, Biochimpharm (Georgia)	Georgian Approval	Topical	Treatment and prophylaxis of bacterial purulent–inflammatory infections (multiple microorganisms)	(199)
Septaphage®table, Septaphage, Phagyo®, PhageStaph, Biochimpharm (Georgia)	Georgian Approval	Oral		
Travelphag™, Biochimpharm (Georgia)	Georgian Approval	Oral	For bacterial infections, indigestion	
*Salmonella* groups A, B, C, D, bacteriophage, Microgen (Russia)	Russian Federation national standard certification	Oral, intrarectal	Treatment and preventative measures for *Salmonella*-related diseases	(195)
*E. coli-Proteus* bacteriophage, Microgen (Russia)	Russian Federation national standard certification	Oral, topical, and intrarectal	Treatment and prevention of enteric and inflammatory disorders that are purulent, dysbacteriosis caused by the bacteria *Proteus*, and enterotoxigenic *E. coli*	
*Klebsiella* purified polyvalent bacteriophage, Microgen (Russia)	Russian Federation national standard certification	Oral, topical, and intrarectal	specific lysis of *K. pneumoniae*, *K. odorifera*, and *K. rhinosclerosis*	
Dysentery polyvalent, bacteriophage, Microgen (Russia)	Russian Federation national standard certification	Oral and intrarectal	specific lysis of the bacterium that causes bacillary dysentery	
Complex Pyobacteriophage, Microgen (Russia)	Russian Federation national standard certification	Oral, topical, and intrarectal	Specific lysis of *E. coli*, *K. pneumoniae*, *Streptococcus*, *Enterococcus*, *Proteus*, and *K. pneumoniae*.	
Bacteriophage dysenteric polyvalent “MediPhag,” Aziya Immunoprepara (Uzbekistan)	Marketed	Oral	A white gelatin capsule with capsules of *Shigella-*fighting bacteriophages that have been dried and frozen.	(200)
LYZODOL®, MB Pharma (Czech Republic)	Marketed	Oral	Against respiratory infections caused by *Propionibacterium acnes*, *Lelliottia amnigena*, *S. aureus*, and *K. pneumoniae*	(201)
Phagogyn, MicroMir (Russia)	Marketed	Topical	A gel containing 74 phages protects against reproductive system bacterial infections.	(202)
Phagodent, MicroMir (Russia)	Marketed	Topical	Contains 72 phage complexes to balance oral microbiota	
Phagoderm, MicroMir (Russia)	Marketed	Topical	A 64-phage skin gel that prevents bacterial skin infections.	
Otophagus, MicroMir (Russia)	Marketed	Topical	A gel comprising 69 phages protects the ear, nose, and throat against bacterial and suppurative inflammation.	
Iskraphage, MicroMir (Russia)	Marketed	Topical	Gel for hygiene and normalization of the skin microbiota.	
Pyofag®, Phagex (Ukraine)	Marketed	Oral and topical	Treatment of pathogenic agents in purulent inflammation and intestinal diseases caused by *P. aeruginosa*, *Proteus vulgaris*, *Proteus mirabilis*, *Streptococcus pyogenes*, *S. aureus*, and *E. coli*.	(172)
Intestifag® polyvalent bacteriophage, Phagex (Ukraine)	Marketed	Oral and topical	Fights *Shigella*, *Salmonella*, *E. coli*, *P. aeruginosa*, *Enterococcus faecalis*, and *S. aureus*-related intestinal diseases.	
BACTELIDE™, Phagelux (China)	FDA-approved IND, preclinical	Transdermal	Patches and sprays for pressure ulcers	(194)
PhageBank, Adaptive Phage Therapeutics (United States)	FDA-approved IND, Phase 1/2	Intravenous	Treat diabetes-related foot osteomyelitis, prosthetic joint infections, chronic recurrent urinary tract infections, eye infections, and lung infections related to cystic fibrosis.	(203)
crPhage™, Locus Biosciences (Korea)	Phase 1b	Injection	Combined with CRISPR-Cas3 to increase the effectiveness of bactericidal treatment for various bacterial infections, including IBD and UTI.	(204)
AcneFree, SciPhage (Republic of Colombia)	Undefined	Transdermal	Fights acne-targeting bacteria	(205)
Balancing Phage Serum, Ellis Day Skin Science (United States)	Marketed	Transdermal	Restore the skin’s microbiome to balance, and eliminate the bacteria that cause blemishes and acne.	(206)
Hydrating Phage Serum, Ellis Day Skin Science (United States)7				

The information and references provided by the companies mentioned above further explain and support the scientific evidence regarding the potential of phage therapy as an antibiotic substitute and the FDA’s recommendations for creating bacteriophage therapy products.

## Limitations and future prospect of implementing phage therapy

While laying out a roadmap of modern phage therapy’s difficulties, it becomes clear that our structured and organized environment brings many obstacles. The first is the uncertain nature of bacteriophages, which are not pure biological macromolecular complexes (like therapeutic proteins) but non-living creatures. As a result, it becomes difficult for regulatory agencies to approve medical therapies or therapeutic substances because legislative approval procedures are intricate, expensive, and time-consuming. Patients may also reject such therapies due to erroneous worries and a lack of knowledge because this approach uses “live viruses” for treatment (207).

Some additional problems attributed to early phage therapy failure and their probable solution and required approach are summarized below:

1. *Narrow host range of phages*: Due to the high specificity of phages, many negative results may have been acquired due to the inability to select lytic phages for the targeted bacterial species. Before utilizing phages for therapeutic purposes, ascertaining the etiologic agent’s susceptibility to phages (208) and using polyvalent phage cocktails that lyse most etiologic agent strains will be a better approach for favorable results.
2. *Insufficient purity of phage preparation*: Early therapeutic phages were in crude lysates of the host bacteria and contained a variety of contaminants (including endotoxins) that may have neutralized the phage’s effects. To create phage preparations with a high purity level, ion-exchange chromatography, high-speed centrifugation, and other advanced purification methods should be utilized (208).
3. *Poor stability and viability of phage preparations*: Commercial phage preparations were supplemented with mercurial, oxidizing agents or heat-treated to ensure bacterial sterility (50). Many of these treatments also may have inactivated the phages, resulting in ineffective phage preparations. Advanced purification techniques can purify phages and ensure they are bacterium-free. The viability and titer of phages should be determined before using them therapeutically.
4. *Lack of understanding of the heterogeneity and mode of action of phages (i.e., lytic vs. lysogenic)*: Some researchers may have used lysogenic phages instead of lytic phages due to their inability to distinguish between them. The temperate phages are not recommended for therapy because they do not “kill” the target bacteria and can cause the transfer of undesired genes. A careful choice should be made when looking for lytic phages. This is essential for preventing potential horizontal gene transfer by lysogenic phages of genes encoding bacterial toxins, antibiotic resistance, etc. (209).
5. *Exaggerated claims of the effectiveness of commercial phage preparation*: One illustration of this would be the Enterophagos, marketed as applicable against herpes infections, urticaria, and eczema (210), diseases that phages could not possibly be successful against. Phage preparations should be provided with detailed, scientifically backed information regarding their effectiveness against specific bacterial pathogens, potential adverse effects, etc.
6. *Failure to establish scientific proof of the efficacy of phage treatment*: Most clinical investigations using therapeutic phages lacked placebo controls (211, 212). Highly pure, lytic phages should be used in well-controlled, double-blinded placebo experiments, and outcomes need to be assessed using patient data and meticulous laboratory testing.
7. *Development of Phage-neutralizing antibodies*: Another issue that could impair the ability of phages to lyse certain bacteria *in vivo* is the emergence of phage-neutralizing antibodies. Indeed, parenteral phage delivery has been associated with the formation of neutralizing antibodies (213). Nevertheless, it is still being determined how substantial of a concern this might be during phage therapy, particularly when administered orally and/or topically. Since the kinetics of phage activity is significantly faster than the host’s synthesis of neutralizing antibodies, the production of neutralizing antibodies should not, in theory, pose a substantial challenge during the initial treatment of acute infections (208). A study by Archana et al. (214) reported the appearance of neutralizing antibodies after the third week following immunization. Complete neutralization of bacteriophages was detectable between 3 and 5 weeks after immunization.
8. *Clearance of phages by reticuloendothelial systems*: According to the study by Merril et al. (215), the reticuloendothelial system’s removal of phages from the patient could represent a problem because it could lower the number of phages to a level that is insufficient to combat the infecting bacteria. The authors chose phages with greater capacity to remain in mice’s bloodstreams using a natural selection process they elegantly referred to as the “serial passage” method. Understanding the mechanisms underlying this characteristic of phages will illuminate critical aspects of how they interact with their bacterial hosts.
9. *The emergence of resistance*: Like antibiotics, bacteria can resist phages over time, leading to the need to develop new phages. Phage therapy’s efficiency may also be constrained by the co-evolution of bacteria and phages, which can result in the emergence of bacterial strains that are resistant to phages (9, 55, 173, 216).
10. *Quality control*: Ensuring the quality and purity of phage preparations can be challenging, as it requires careful monitoring of phage production and purification processes (217, 218).
11. *Delivery methods*: The mode of administration can impact the effectiveness of phage therapy since enough phages must be delivered to the infection site for treatment to be effective. This can be challenging in some cases, such as treating deep tissue infections or infections in areas that are difficult to access (22, 24).

Many phages produce virulence factors or toxins, making them ineffective as antimicrobial agents. The precise elimination of potentially dangerous genetic information from viral genomes can tackle this crucial safety concern. The release of bacterial toxins, lipopolysaccharides, and other pathogen-associated molecular patterns (PAMPs) as a result of phage-induced lysis might trigger an innate immune response, increase virulence, or result in additional harm. To lessen these possibly detrimental effects, phage variants that are engineered to destroy target cells without releasing PAMP can be utilized. Phages can be designed to cleave defined nucleotide sequences and genotypes, for example, host cells that carry antibiotic-resistance genes or particular virulence factors, by delivering sequence-specific CRISPR-Cas nucleases via modified genomes or phagemids. Phagemids or modified genomes can be used to deliver sequence-specific CRISPR-Cas nucleases, which can then be programmed to cleave specific genotypes and nucleotide sequences (217).

Phage therapy has not yet been widely adopted in clinical practice, despite increased interest in phages and the collaborative efforts of scientists and clinicians that have led to increased case reports. The fundamental reason for this lack of implementation is the ongoing dearth of reliable clinical evidence on phage therapy, which prevents ethical, actuarial, and governmental authorities from addressing their concerns. To make phage therapy an integral part of clinical practice in the future, a focused and practical regulatory framework for personalized phage therapy techniques and interdisciplinary collaboration between researchers, microbiologists, clinicians, and pharmacists are essential.

## Conclusion

Phage therapy holds the promise of unveiling novel approaches to combat bacterial infections. Particularly noteworthy is the potential of phage cocktails, which could revolutionize the treatment landscape by offering solutions for a wide spectrum of bacterial diseases that have proven resistant even to the latest generations of antibiotics. Nevertheless, several pivotal challenges must be surmounted to harness the full potential of phages as antibacterial agents. These challenges encompass ensuring safety, evaluating effectiveness, and assessing the likelihood of immune responses triggered by administered phages. Additionally, refining phage purification techniques and optimizing their growth are essential hurdles that demand attention.

Within clinical settings, phage therapy has emerged as a beacon of promise, despite the obstacles. Ongoing technological advancements and strides in genetic engineering hold the potential to unlock the creation of more precise and potent phages. The synergistic use of phage therapy in conjunction with other therapeutic modalities, such as immunity modifiers or antibiotics, may further enhance its efficacy. Beyond the realm of human healthcare, phage therapy extends its reach into veterinary medicine, agriculture, and food safety, presenting multifaceted applications with vast potential.

In summation, phage therapy stands as a formidable contender in the battle against antibiotic-resistant bacteria, offering a compelling alternative or adjunct to traditional antibiotic treatments. Nevertheless, these prospects are not without their challenges. Continued research and development efforts are imperative to fully exploit the myriad advantages this promising technology offers and to surmount the remaining obstacles that lie in its path. The journey to harness the full potential of phage therapy is still underway, but the destination promises a brighter future in the fight against bacterial infections.

## Author contributions

SK, MG, and GN planned to compile phage data systematically. SK, RK, and MG collected and analyzed the data from various authentic resources. SK compiled the data and finalized the review article as per the proposed objectives from the database and the major contributor to writing the manuscript. SK, MG, SB, and GN finalized the draft, and the published data was presented in the review article. All authors have read and approved the final manuscript.
